# RETRACTED: Ahmed et al. Omega-3 Self-Nanoemulsion Role in Gastroprotection against Indomethacin-Induced Gastric Injury in Rats. *Pharmaceutics* 2020, *12*, 140

**DOI:** 10.3390/pharmaceutics16020192

**Published:** 2024-01-29

**Authors:** Osama A. A. Ahmed, Usama A. Fahmy, Rana Bakhaidar, Mohamed A. El-Moselhy, Solomon Z. Okbazghi, Al-Shaimaa F. Ahmed, Asmaa S. A. Hammad, Nabil A. Alhakamy

**Affiliations:** 1 Department of Pharmaceutics, Faculty of Pharmacy, King Abdulaziz University, Jeddah 21589, Saudi Arabia; uahmedkauedu.sa@kau.edu.sa (U.A.F.); rbakhaidar@kau.edu.sa (R.B.); nalhakamy@kau.edu.sa (N.A.A.); 2 Department of Pharmaceutics & Industrial Pharmacy, Faculty of Pharmacy, Minia University, Minia 61519, Egypt; 3 Department of Pharmacology, School of Pharmacy, Ibn Sina National College, Jeddah 22413, Saudi Arabia; m_moselhy64@yahoo.com; 4 Department of Pharmacology and Toxicology, Faculty of Pharmacy, Minia University, Minia 61519, Egypt; shaimaa.faissal@minia.edu.eg (A.-S.F.A.); asmaa.soli@yahoo.com (A.S.A.H.); 5 Global Analytical and Pharmaceutical Development, Alexion Pharmaceuticals, New Haven, CT 06510, USA; solomon.z.okbazghi@gmail.com

The journal retracts the article, “Omega-3 Self-Nanoemulsion Role in Gastroprotection against Indomethacin-Induced Gastric Injury in Rats” [1], cited above. 

Following publication, concerns were brought to the attention of the publisher regarding incorrect images and image overlap with later publications.

Adhering to our complaint’s procedure, an investigation was conducted that confirmed the presence of an incorrect image (Figure 4, panel B) and the partial and full overlap of this image within later publications [2,3], listing different experimental conditions. 

While the authors fully cooperated with the Editorial Office during the investigation, they were unable to satisfactorily explain the mistaken inclusion of this image within this article [1], nor were they able to meet the required quality standards of raw images in order to consider a correction as per the journals original image requirements policy (https://www.mdpi.com/journal/pharmaceutics/instruc-tions#oriimages). As a result, the Editorial Board and Editor-in-Chief were unable to confirm the reliability of the overall findings and subsequently decided to retract this paper.

This retraction was approved by the Editor-in-Chief of the journal *Pharmaceutics*. 

The authors did not agree to this retraction. 
